# Supplementary material for: Reduced macular vessel density and inner retinal thickness correlate with the severity of cerebral autosomal dominant arteriopathy with subcortical infarcts and leukoencephalopathy (CADASIL)
Source: PLoS One. 2022 May 26;17(5):e0268572. doi: 10.1371/journal.pone.0268572 (PMC9135286; doi:10.1371/journal.pone.0268572)
Supplement: Attachment — Submitted filename: Response to Reviewer.docx [file pone.0268572.s002.docx]

**Response to Reviewer’s Comments**

We thank the reviewer for pointing out the suggestions to make the manuscript better. Please kindly see the following point-by-point response. In the revised manuscript, any change or addition of the words, paragraphs, or figures are highlighted in yellow. We hope this revision would substantially strengthen our manuscript.

Reviewer’s Comments: This manuscript addresses an important need for non-invasive methodologies to diagnose and track the progression of CADASIL. I thought the experiments were well designed and this is the most detailed OCT-A evaluation I have so far seen for this disease population including appropriate controls. The results and shown correlations provide a strong case for the use of this technique for this disease population.

**Point 1**: In order to improve the paper, I would suggest that some figures to display the correlations (bar graphs) might be helpful when following the text to help readers.

**Response 1**: Thank you for the suggestion. We have added a figure (**Figure 3**) to display the correlations between parameters of OCT-A and cognition, gait, and neuroimaging markers. We used a forest plot to display the multiple correlations estimates and their confidence intervals. Those with significant correlations (p<0.05) were mentioned with text next to it. We hope this figure can be helpful for the readers.

**Point 2**: I would also suggest a schematic of the eye for the several measured regions to improve clarity.

**Response 2**: Thank you for the suggestion. We have added a figure (**Figure 1**) to show the several pre-defined measured regions of the eye. We hope this figure can also be helpful for the readers.

Point 3: Lastly, the English in the manuscript is good but I would have a native English speaker (if possible) check the text for a few areas where the language isn't clear, particularly in the introduction. For example, CADASIL does not "mainly affect middle-aged adults". It actually affects a wide range of ages but disease onset is generally around the 3rd decade of life.

**Response 3**: Thank you for your thoughtful reading. This manuscript has been edited by an Academic Editing company (shown in the following) before submission. Nevertheless, we acknowledged there were still some places for improvement. Therefore, we’ve re-written some parts of the manuscript, including the introduction part. Hope this will improve our manuscript.
